# Quantification of active mitochondrial permeability transition pores using GNX-4975 inhibitor titrations provides insights into molecular identity

**DOI:** 10.1042/BCJ20160070

**Published:** 2016-04-26

**Authors:** Andrew P. Richardson, Andrew P. Halestrap

**Affiliations:** * School of Biochemistry and Bristol CardioVascular, University of Bristol, Medical Sciences Building, University Walk, Bristol BS8 1TD, U.K.

**Keywords:** adenine nucleotide translocase, calcium, inhibition, mitochondrial permeability transition pores, mitochondrial phosphate carrier, molecular mechanism

## Abstract

Inhibition of the mitochondrial permeability transition pore (MPTP) by the novel inhibitor GNX-4975 was characterized. Titration of MPTP activity in de-energized rat liver mitochondria allowed determination of the number of GNX-4975-binding sites and their dissociation constant (*K*_i_). Binding sites increased in number when MPTP opening was activated by increasing [Ca^2+^], phenylarsine oxide (PAO) or KSCN, and decreased when MPTP opening was inhibited with bongkrekic acid (BKA) or ADP. Values ranged between 9 and 50 pmol/mg of mitochondrial protein, but the *K*_i_ remained unchanged at ∼1.8 nM when the inhibitor was added before Ca^2+^. However, when GNX-4975 was added after Ca^2+^ it was much less potent with a *K*_i_ of ∼140 nM. These data imply that a protein conformational change is required to form the MPTP complex and generate the GNX-4975-binding site. Occupation of the latter with GNX-4975 prevents the Ca^2+^ binding that triggers pore opening. We also demonstrated that GNX-4975 stabilizes an interaction between the adenine nucleotide translocase (ANT), held in its ‘c’ conformation with carboxyatractyloside (CAT), and the phosphate carrier (PiC) bound to immobilized PAO. No components of the F_1_F_o_-ATP synthase bound significantly to immobilized PAO. Our data are consistent with our previous proposal that the MPTP may form at an interface between the PiC and ANT (or other similar mitochondrial carrier proteins) when they adopt novel conformations induced by factors that sensitize the MPTP to [Ca^2+^]. We propose that GNX-4975 binds to this interface preventing a calcium-triggered event that opens the interface into a pore.

## Abstract (precis)

The molecular identity of the mitochondrial permeability transition pore (MPTP), a key player in cell death, remains controversial. Here we use a novel MPTP inhibitor to demonstrate that formation of the pore involves native mitochondrial membrane proteins adopting novel conformations.

## INTRODUCTION

The mitochondrial permeability transition pore (MPTP) is a large non-specific channel in the inner mitochondrial membrane (IMM) whose opening is triggered by high matrix [Ca^2+^] to which it can be sensitized by a variety of factors. These include elevated [P_i_], adenine nucleotide depletion, agents such as carboxyatractyloside (CAT) that stabilize the adenine nucleotide translocase (ANT) in its cytoplasmic facing (‘c’) conformation, mild chaotropic agents such as KSCN and oxidative stress or its chemical mimic phenylarsine oxide (PAO) [1]. It is now widely accepted that MPTP opening plays a central role in the cell death that underlies several pathological conditions including ischaemia/reperfusion injury [2–5]. However, the exact molecular composition of the MPTP remains unresolved [1,4,6,7]. There is general agreement that the matrix peptidyl prolyl *cis*–*trans* isomerase cyclophilin D (CyP-D) acts as key regulator of the MPTP, enhancing its sensitivity to [Ca^2+^]. Indeed, CyP-D is the site of action of two potent inhibitors of MPTP opening, cyclosporin A (CsA) and sanglifehrin A (SfA), both of which protect cells from death mediated by MPTP opening such as in ischaemia/reperfusion injury [3]. However, the molecular composition of the IMM components of the MPTP, with which CyP-D interacts, remains controversial.

Studies from our and other laboratories have implicated the ANT and the phosphate carrier (PiC), both of which can bind CyP-D [8–11] and whose ligands modulate MPTP opening [10,12–14]. Indeed the ANT, when reconstituted into phospholipid membranes, does demonstrate calcium-activated pore formation that can be modulated by CyP-D [9,15,16]. However, knockdown of either the PiC or ANT does not prevent MPTP opening, although the response to calcium and ANT ligands is severely attenuated [11,17,18]. More recently, others have proposed a role for the F_1_F_o_-ATP synthase which can also interact with CyP-D [19], although there are competing hypotheses as to which subunits are involved. Data from the laboratories of Pinton [20] and Jonas [21] have implicated the c subunits of the F_o_ ATP synthase, whereas Bernardi and colleagues propose a role for dimers of the F_1_F_o_-ATP synthase, perhaps involving subunits associated with the c-ring in the membrane such as a, e, f, g and A6L moving to produce the pore [4,22]. We and others have expressed reservations about such a role for the F_1_F_o_-ATP synthase since a considerable body of evidence is better explained by interactions of pore regulators with the ANT and PiC [1,6,23]. Nevertheless, it remains possible that an interaction between the ANT, PiC and ATP synthase is involved in MPTP formation and such interactions may occur in the ATP synthasome [1,6,24,25]. Most recently evidence has been presented for the mitochondrial AAA protease SPG7 (spastic paraplegia 7) being essential for MPTP opening [26], although others have questioned these conclusions [27].

The role of CyP-D in mediating MPTP opening was first demonstrated in our laboratory by performing CsA inhibitor titrations of MPTP opening and the peptidyl prolyl *cis*–*trans* isomerase activity of CyP-D. These demonstrated that the *K*_i_ for CsA inhibition of both actions was the same (∼5 nM) and that the high affinity of binding allowed calculation of the number of CsA-binding sites which were also identical for both processes [28,29]. Recently, Congenia has developed a new class of highly potent cinnamic anilide**-**based inhibitors of MPTP opening that protect against ischaemia/reperfusion injury of the heart [30] and amyotrophic lateral sclerosis [31]. Although the generic structure of these inhibitors is available [31,32], the exact structure of GNX-4975 is not in the public domain. MPTP inhibition by these agents does not involve CyP-D, implying that they must interact with another component of the MPTP. In the present study, we explore the binding site of the inhibitors in more detail using GNX-4975 which we demonstrate to have a *K*_i_ for MPTP inhibition of ∼2 nM under a variety of conditions. Importantly, we show that the number of binding sites for GNX-4975 increases when MPTP opening is activated by PAO or KSCN, and decreases when MPTP opening is inhibited with bongkrekic acid (BKA) or ADP. These data imply that a protein conformational change is required to form the MPTP and that this generates the binding site for GNX-4975. We further demonstrate that GNX-4975 enhances the association of ANT to the PAO-bound PiC. This is consistent with our previous proposal that an interface between the PiC and ANT may occur when these proteins adopt novel conformations induced by factors that sensitize the MPTP to [Ca^2+^] [1]. We propose that GNX-4975 binds to this interface preventing a calcium-triggered event that opens the interface into a pore.

## EXPERIMENTAL

### Materials

Unless otherwise stated, all chemical and biochemicals were obtained from Sigma–Aldrich or Fisher Scientific. Antibodies against the C-terminus of the ANT and PiC were raised either in-house or commercially as described in [10]. The use of rats conformed to the U.K. Animals (Scientific Procedures) Act 1986 and was approved by the appropriate University of Bristol ethics committee (UB/09/012).

Mitochondria were prepared from livers of 250–275 g male Wistar rats by Dounce Potter homogenization in sucrose isolation buffer (ISB: 300 mM sucrose, 10 mM Tris/HCl and 2 mM EGTA, pH 7.4) followed by differential centrifugation and Percoll® density-gradient centrifugation as described previously [28]. Preparation of beef and pig heart mitochondria involved initial tissue disruption with a Polytron 10-35 GT homogenizer [10] with subsequent steps being the same as for liver mitochondria. For measurement of MPTP opening in de-energized rat liver mitochondria, they were routinely prepared 1 day before they were used to assay MPTP opening and stored overnight on ice. This avoided changes to the [Ca^2+^] sensitivity of the MPTP caused by progressive loss of total adenine nucleotides over the first few hours of storage on ice [12,32].

### Methods

#### Measurement of MPTP opening in de-energized mitochondria

This was performed by one of two techniques. A swelling assay was used in which pore opening was triggered by addition of Ca^2+^ [12,28]. In addition a shrinkage assay was employed in which pre-swollen mitochondria were incubated with the required [Ca^2+^] to open the MPTP, the extent of which was then determined by the rate of shrinkage induced by addition of polyethylene glycol (PEG) [32]. In both cases the initial rate of decrease (swelling) or increase (shrinkage) in light scattering (monitored as *A*_520_) was used to determine the extent of MPTP opening. For the swelling assay, mitochondria were incubated at 25°C and 0.5–1 mg of protein/ml in de-energized assay buffers containing either 150 mM KSCN or 125 mM KCl plus 2.5 mM potassium phosphate as the osmotic support and 20 mM MOPS, 10 mM Tris/HCl, 2 mM nitrilotriacetic acid (NTA), 0.5 μM rotenone, 0.5 μM antimycin A and 2 μM A23187 at pH 7.2. MPTP opening was initiated by addition of [Ca^2+^] (usually 50 μM free) and *A*_520_ was monitored continuously in a spectrophotometer with computerized data acquisition. When GNX-4975 was included, it was added 2 min prior to the addition of Ca^2+^, whereas incubations with other drugs or reagents were carried out for 4 min prior to the addition of Ca^2+^.

For the shrinkage assay, mitochondria (2 mg/ml) were pre-swollen by incubation for 20 min at 30°C in standard de-energized KSCN buffer (as above) but without added NTA or A23187 and with addition of 1 mM CaCl_2_. Any residual swelling was terminated by an addition of 1.2 mM EGTA, which also resealed swollen mitochondria. The resulting swollen mitochondria were collected by centrifugation at 12 000 ***g*** for 10 min and resuspended at 2 mg/ml in de-energized KSCN buffer without added NTA or A23187. In order to ensure equilibrium of matrix with the buffer, the swollen mitochondria were incubated again at 30°C supplemented with 1 mM CaCl_2_. After 3 min, 1.2 mM EGTA was added to reseal the mitochondria before centrifugation at 12 000 ***g*** for 10 min. The swollen mitochondria were then resuspended at 30 mg/ml in de-energized KSCN buffer containing 2 mM NTA and 2 μM A23187 and stored on ice. The extent of MPTP opening in these pre-swollen mitochondria was determined following incubation at 0.33 or 0.67 mg/ml for the stated time in KSCN or KCl buffer containing, where indicated, the required [GNX-4975] and free [Ca^2+^] (calculated as described in [33]). Shrinkage was initiated by rapid addition of 0.5 ml of 50% (w/v) PEG (to give a final PEG concentration of 7%, w/v) and was continuously monitored (ten data points per second) as an increase in *A*_520_.

#### Determination of the number of binding sites and *K*_i_ for GNX-4975 inhibition of the MPTP

The initial rate of swelling or shrinking of mitochondria pre-incubated with increasing concentrations of GNX-475 was determined by differentiation of the time course of *A*_520_ change. As described previously [34,35], the data were then fitted using FigSys (BioSoft) to the equation for the inhibition of rate by a tight binding inhibitor:

$$V=\frac{k\cdot E_{t}\cdot P-k\cdot \left(B-\sqrt{B\cdot B-4\cdot C}\right)}{2}$$

$$\mathrm{where}B=E_{t}\cdot P+I+K_{i}$$

$$\mathrm{and}\;C=E_{t}\cdot P\cdot I$$

*E*_t_ represents the number of GNX-4975-binding sites in pmol/mg, *P* is the mitochondrial protein concentration (mg/ml), *K*_i_ is the dissociation constant for GNX-4975 (nM) and *k* is the rate constant for swelling (∆*A*_520_·s^−1^·nM^−1^). Unless otherwise stated, for each titration, a separate *E*_t_, *K*_i_ and *k* were derived and the mean value ± S.E.M. calculated for several mitochondrial preparations (*n*=3–6 as indicated). In order to correct for variations in the rates of swelling or shrinking determined using different mitochondrial preparations on different days, rates at any particular inhibitor concentration were usually calculated as a percentage of the maximal rate of swelling in the absence of inhibitor or other MPTP modulating reagents. In this case, the *k* value becomes 100/*E*_t_ and so its value is not presented.

#### Measurement of MPTP opening in energized mitochondria

This was performed by simultaneously measuring extra-mitochondrial [Ca^2+^] with Fura-FF fluorescence, membrane potential with Rhodamine 123 fluorescence and mitochondrial swelling with light scattering at 490 nm as described previously [14]. The incubation medium consisted of 125 mM KCl, 10 mM Tris/HCl, 20 mM MOPS, 2.5 mM potassium phosphate, 5 mM L-glutamate, 2 mM L-malate, 20 μM EGTA, 0.1 μM Fura-FF and 100 nM Rhodamine-123, pH 7.2, at 30°C. MPTP opening was induced by sequential additions of Ca^2+^ as indicated in the figures.

#### Preparation and solubilization of inner mitochondrial membranes

Centrifugation steps in this process were carried out using a benchtop Eppendorf 5415R centrifuge at 4°C. Mitochondria were washed in ISB and centrifuged at 12 000 ***g*** for 5 min before re-suspension in ISB containing protease inhibitors (4 μg/ml each of antipan, pepstatin A and leupeptin plus 0.5 mM benzamidine and 0.5 mM PMSF). Mitochondria were incubated with digitonin (0.12 mg/mg of mitochondrial protein) at 4°C for 15 min on a rotary mixer, and the resulting mitoplasts collected by centrifugation at 9000 ***g*** for 10 min before resuspension in ISB containing protease inhibitors and PEG ether W1 (Sigma P7516, formerly known as Lubrol) at 0.16 mg/mg of mitochondrial protein. The mixture was incubated at 4°C for 15 min on a rotary mixer and insoluble material removed by centrifugation at 4000 ***g*** for 30 s. The supernatant was centrifuged at 125 000 ***g*** and 4°C for 30 min in a Beckman Optima™ TLX ultracentrifuge (TLA-55 rotor) to yield the IMM pellet. Where required, pre-treatment of mitochondria with drugs or reagents was performed in ISB at a protein concentration of 2 or 5 mg/ml for 10 min at room temperature with constant agitation. The treated mitochondria were collected by centrifugation at 12 000 ***g*** for 5 min and re-suspended in ISB containing protease inhibitors (as above), prior to digitonin treatment. Where indicated, drugs or reagents were present throughout the duration of the isolation, including the Lubrol treatment.

#### Binding of solubilized IMM proteins to immobilized phenylarsine oxide

An immobilized PAO matrix was synthesized by coupling 4-aminophenylarsine oxide to Affi-gel 10 (Bio-Rad Laboratories 153-6099) as described previously [10,12]. The resin was washed twice in 10 volumes of PAO column buffer (PCB: 150 mM Na_2_SO_4_, 50 mM HEPES and 1 mM EGTA, pH 7.2) containing 0.5% (v/v) Triton X-100 and then resuspended to give a 50% (v/v) slurry ready for use. IMM fractions from mitochondria incubated with the required reagents were resuspended in PCB containing 1% (v/v) Triton X-100 and protease inhibitors (as above) and solubilized for 1 h on a rotary mixer at 4°C. Insoluble material was removed by centrifugation at 16 000 ***g*** for 12 min and the supernatant transferred to prepared aliquots of pre-washed 50% slurry of PAO beads (200 μl/1 per mg of solubilized protein) and in-cubated for 1 h on a rotary mixer at 4°C. Beads were collected by centrifugation at 200 ***g*** for 1 min and were washed five times with 1 ml of PCB containing 3% (v/v) Triton X-100 to remove non-specifically bound proteins. Specifically bound proteins were then eluted by incubating in 80 μl of 25 mM DTT in PCB containing 1% (v/v) Triton X-100 for 30 min on a rotary mixer at 4°C. Beads were collected by centrifugation at 2000 ***g*** for 3 min and the supernatant (70 μl) was added to an equal volume of SDS/PAGE sample buffer. Samples were analysed by SDS/PAGE and Western blotting or analysed by Orbitrap MS in the University of Bristol Proteomics Facility. Development of Western blots was performed using ECL with film detection of the emitted light and analysis of the scanned film with ImageJ software (http://imagej.nih.gov/). The integrated optical density for each band was calculated as the product of the ‘area’ and ‘mean grey value’ with subtraction of a background signal obtained for an equivalent adjacent area lacking any band. In one set of experiments comparative analysis was performed using direct detection of ECL with an ODDESEY Fc (LI-COR) and analysis of the bands performed using Image Studio (LI-COR). The band intensities were determined by the software and corrected for background by subtracting the mean signal of five pixels above and below each band of interest.

## RESULTS

### GNX-4975 is a tight binding inhibitor of the MPTP

We first confirmed that GNX-4975 is a potent inhibitor of MPTP opening in energized liver mitochondria by simultaneously measuring extra-mitochondrial [Ca^2+^] (Fura-FF fluorescence), membrane potential (Rhodamine 123 fluorescence) and mitochondrial swelling (light scattering at 490 nm) as described previously [14]. As shown in Supplementary Figure S1, sequential additions of Ca^2+^ were taken up by the mitochondria until the MPTP opened, at which time the accumulated Ca^2+^ was released, the membrane potential was dissipated and the light scattering rapidly decreased as the mitochondria swelled. In control mitochondria, this occurred after addition of 70–80 μM Ca^2+^, whereas in the presence of 0.2 μM GNX-4975 MPTP opening required 200–240 μM Ca^2+^.

**Figure 1:**
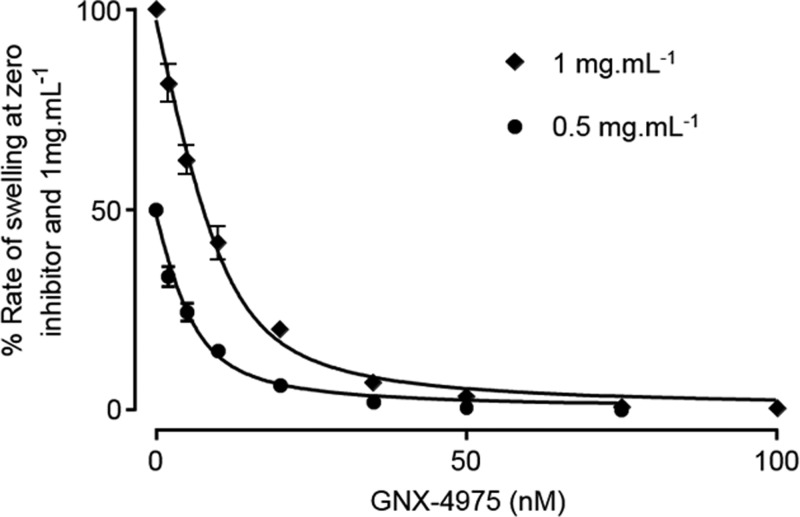
GNX-4975 is a tight binding inhibitor of the MPTP Opening of the MPTP in isolated aged liver mitochondria under de-energized conditions in KSCN medium was induced by 50 μM Ca^2+^ and the initial rate of change in *A*_520_ was determined as described in the Experimental section. For each concentration of GNX-4975, two protein concentrations were employed (0.5 and 1 mg/ml) and rates of swelling were expressed as a percentage of the zero inhibitor rate for which the value at 0.5 mg/ml protein was normalized to 50% of that at 1 mg of protein to overcome the lack of linearity of *A*_520_ with protein concentrations. Data are shown as means ± S.E.M. (error bars) for six different mitochondrial preparations, each of which was fitted to the tight binding inhibitor equation to calculate the number of inhibitor-binding sites (*E*_t_) and their *K*_i_ value. The fitted line was calculated using the derived mean values (±S.E.M.) for *E*_t_ (12.6±1.7 pmol/mg) and *K*_i_ (1.95±0.17 nM).

In order to determine an accurate concentration-dependence of MPTP inhibition by GNX-4975, we employed de-energized mitochondria in the presence of a Ca^2+^ ionophore (A23187), since this avoids any complications that might be caused by secondary effects of the drug on mitochondrial energization or calcium transport. We also performed the assays in medium containing 150 mM KSCN since we have previously shown that this mildly chaotropic buffer promotes the active conformation of the MPTP and generates very consistent data [32]. GNX-4975 was added at the required concentration to the mitochondrial suspension 2 min before Ca^2+^ was added to initiate MPTP opening whose extent was determined from the initial rate of swelling (decrease in *A*_520_) quantified by taking the first derivative of each time course. Figure 1 shows mean data (±S.E.M.) for such inhibitor titrations on six separate mitochondrial preparations each used at two different concentrations of mitochondria (0.5 and 1 mg of protein/ml). It is immediately apparent that more GNX-4975 was required to give 50% inhibition at the higher protein concentration as is predicted for a very tight binding inhibitor whose *K*_i_ value is less than the concentration of binding sites. This was observed previously for CsA inhibition of MPTP opening and using the appropriate equation for inhibition by a tight binding inhibitor this allowed calculation of the true *K*_i_ (dissociation constant) for inhibitor binding and the number of inhibitor-binding sites (*E*_t_) [28,29]. We successfully analysed the data of Figure 1 in this way and determined *E*_t_ to be 12.6 ± 1.7 pmol/mg and the dissociation constant for drug binding (*K*_i_) to be 1.95 ± 0.17 nM (values presented as means ± S.E.M. for six separate mitochondrial preparations). It should be noted that absolute values of light scattering are not linearly related to mitochondrial protein concentration and can also vary from day to day. Thus, to analyse the data from several experiments, the rates of swelling in the absence of inhibitor at 0.5 and 1 mg/ml mitochondrial protein were set at 50 and 100 respectively and values at each concentration of GNX-4975 were calculated relative to the control value.

### MPTP activation increases the number of GNX-4975-binding sites without affecting their *K*_i_

**Figure 2:**
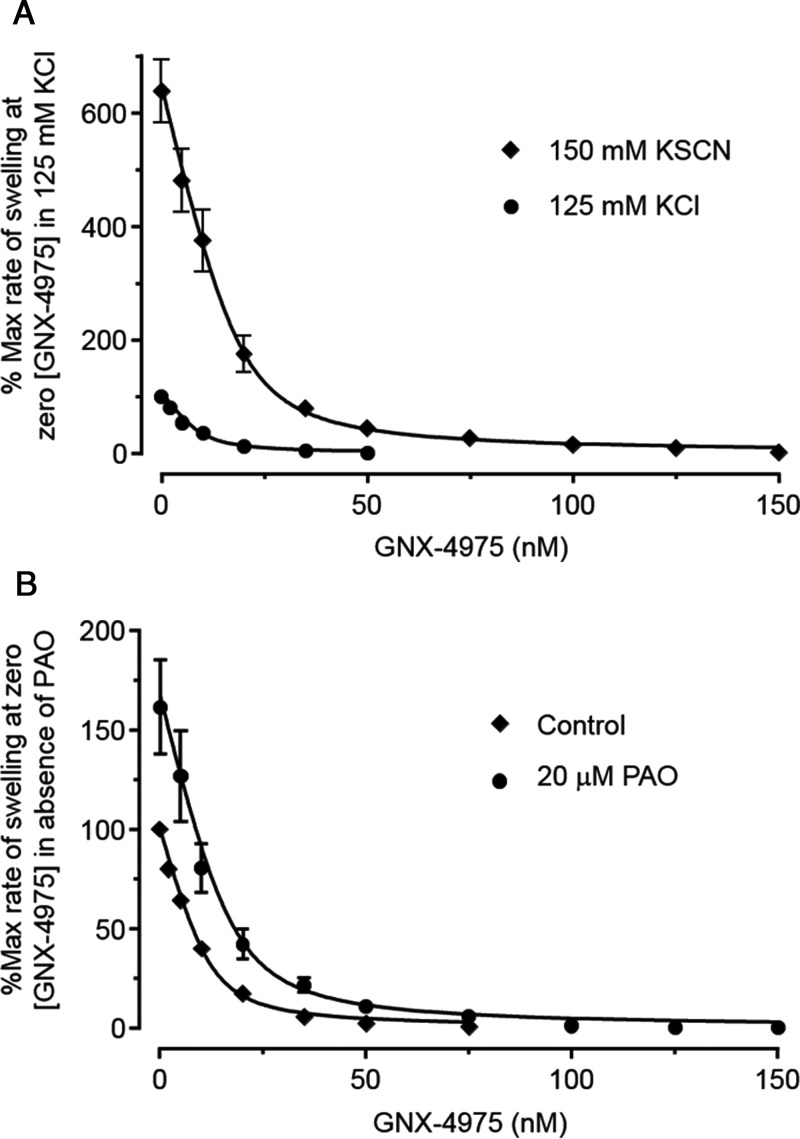
Increasing the propensity of the MPTP to open with either KSCN buffer or PAO increases the number of GNX-4975-binding sites Opening of the MPTP was measured as described in Figure 1 but for each separate mitochondrial preparation a single protein concentration (1 mg/ml) was employed. In (**A**), parallel measurements were made using either the standard 150 mM KSCN medium or one in which the KSCN was replaced with 125 mM KCl+2.5 mM potassium phosphate. For both media, MPTP opening was initiated by addition of 150 μM Ca^2+^ and rates of swelling were expressed as a percentage of the zero inhibitor rate in the KCl medium. Mean data ± S.E.M. (error bars) for five separate mitochondrial preparations are given. Data were fitted as described in Figure 1 and generated mean derived values (±S.E.M., *n*=5) for *E*_t_ of 9.43±1.4 and 19.2±2.2 pmol/mg in KCl and KSCN medium respectively (*P* ≤ 0.01 calculated using the unpaired Student's *t* test). The corresponding calculated *K*_i_ values were 1.68±0.13 and 2.29±0.17 nM respectively. In (**B**), mitochondria were either untreated (control) or pre-incubated with 20 μM PAO for 3 min before addition of 50 μM Ca^2+^ in KSCN medium to initiate swelling. Mean data ± S.E.M. (error bars) for six separate mitochondrial preparations are given. The rate of swelling in the absence of both PAO and GNX-4975 was set at 100 and all other rates calculated relative to this. Data, fitted as in (**A**) gave mean values (±S.E.M.) for *E*_t_ in the absence and presence of PAO of 11.9±0.92 and 17.2±1.3 pmol/mg respectively (*P*<0.01) with corresponding *K*_i_ values of 1.93±0. 21 and 2.56±0.14 nM.

The data shown in Figure 2A compare the inhibition profile of GNX-4975 in KSCN medium, which activates the MPTP, with data obtained in a more physiological KCl-based medium containing 125 mM KCl and 2.5 mM potassium phosphate. It is immediately apparent that rates of swelling at zero inhibitor are less in the KCl medium (set at 100%) than in KSCN medium, whereas the derived *K*_i_ values (mean ± S.E.M. for five separate experiments) were similar, being 1.68±0.13 and 2.29±0.17 nM respectively. However, the calculated number of GNX-4975-binding sites in the KSCN buffer (19.2±2.2 pmol/mg) was significantly higher (*P*<0.01) than in the KCl buffer (9.43±1.4 pmol/mg). It should be noted that in these experiments, in order to ensure that rates of swelling in KCl medium were sufficiently rapid to allow accurate inhibitor titrations, we activated MPTP opening with 150 μM Ca^2+^. As discussed further below, this explains the higher value of *E*_t_ than determined in Figure 1 where only 50 μM Ca^2+^ was employed. These data suggest that activation of the MPTP by KSCN involves an increase in the active conformation of the pore to which GNX-4975 binds. It should be noted that it is not possible to relate the rates of swelling directly to the number of binding sites because many additional factors can affect the absolute value of *A*_520_ as noted above.

**Figure 3:**
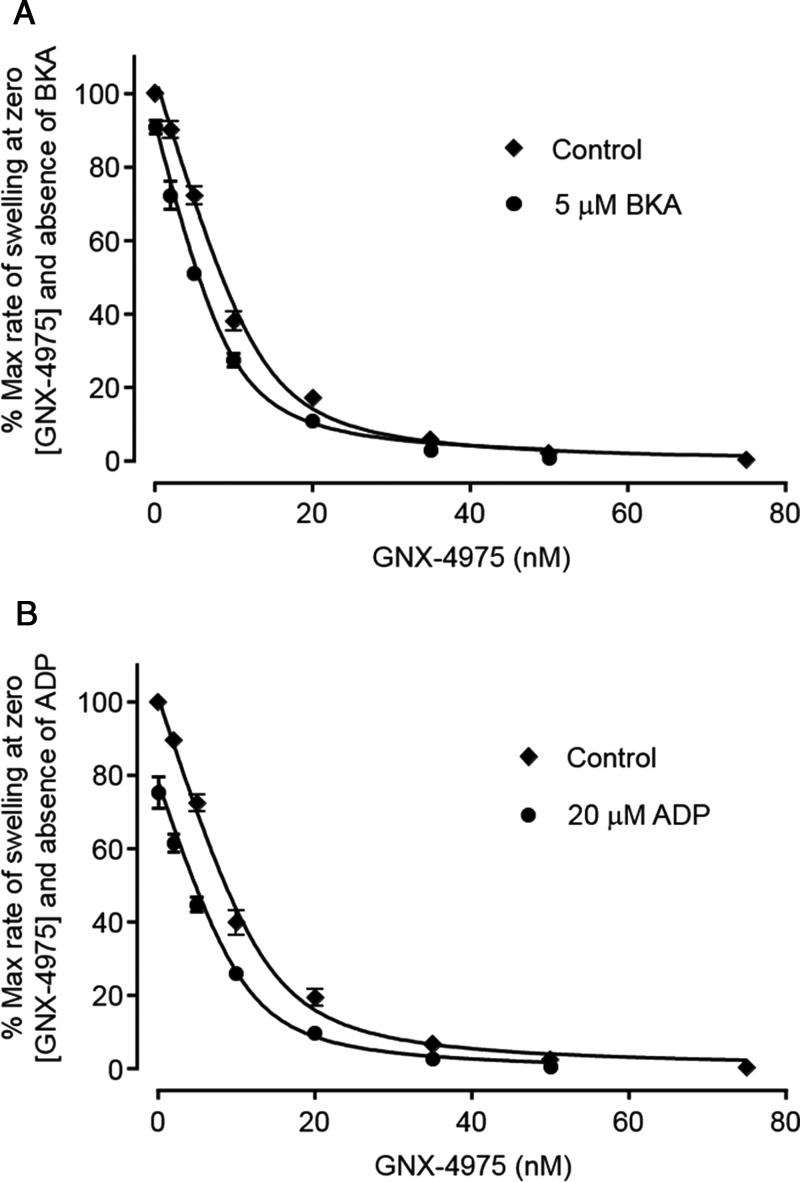
The ‘m’ conformation of the ANT reduces the number of GNX-4975-binding sites Experiments were performed exactly as described for Figure 2B but with 3 min of pre-treatment of either 5 μM BKA (**A**) or 20 μM ADP (**B**) prior to addition of 50 μM Ca^2+^ to initiate swelling. Mean data ± S.E.M. (error bars) for six separate mitochondrial preparations are given. The rate of swelling in the absence of both GNX-4975 and ADP or BKA was set at 100 and all other rates calculated relative to this. Data were fitted as described in Figure 1. In (**A**) mean values (±S.E.M.) for *E*_t_ in the absence and presence of BKA were 13.2±0.64 and 9.43±0.47 pmol/mg (*P*<0.001), whereas *K*_i_ values remained unchanged (1.45±0.23 and 1.46±0.16 nM respectively). In (**B**), the derived values for *E*_t_ in the absence and presence of ADP were 14.1±0.83 and 11.0±0.97 pmol/mg (*P* < 0.05) with *K*_i_ values of 1.57±0.24 and 1.45±0.15 nM respectively.

Activation of the MPTP can also be achieved by exposing the mitochondria to oxidative stress and this can be mimicked by the vicinal thiol reagent PAO [12,36], which acts, at least in part, by cross-linking thiol groups on the ANT and PiC [10,12,37]. In Figure 2B we show that, following a short pre-incubation with 20 μM PAO, MPTP opening was activated as expected and that this led to an increase in the number of binding sites for GNX-4975 from 11.9±0.92 to 17.2±1.3 pmol/mg (means ± S.E.M. for six separate mitochondrial preparations; *P*<0.01) without any significant change in *K*_i_ (1.93±0.21 and 2.56±0.14 nM respectively). Conversely, BKA, an inhibitor of the ANT that traps the carrier in its matrix facing (‘m’) conformation, inhibits MPTP opening as does ADP which also favours the ‘m’ conformation [12,13,38,39]. In Figure 3A, we show that mitochondrial pre-treatment with 5 μM BKA decreased the number of GNX-4975-binding sites from 13.2±0.64 to 9.43±0.47 pmol/mg (means ± S.E.M. for six separate mitochondrial preparations; *P*<0.001), again without any change in *K*_i_ (1.45±0.23 and 1.46±0.16 nM respectively). In Figure 3B, we show similar data for mitochondria pre-treated with 20 μM ADP which decreased the number of GNX-4975-binding sites from 14.1±0.83 to 11.0±0.97 pmol/mg (means ± S.E.M. for six separate mitochondrial preparations; *P*<0.05), also without any change in *K*_i_ (1.57±0.24 and 1.44±0.15 nM respectively).

### The number of GNX-4975-binding sites is increased when higher [Ca^2+^] is used to induce MPTP opening

**Figure 4:**
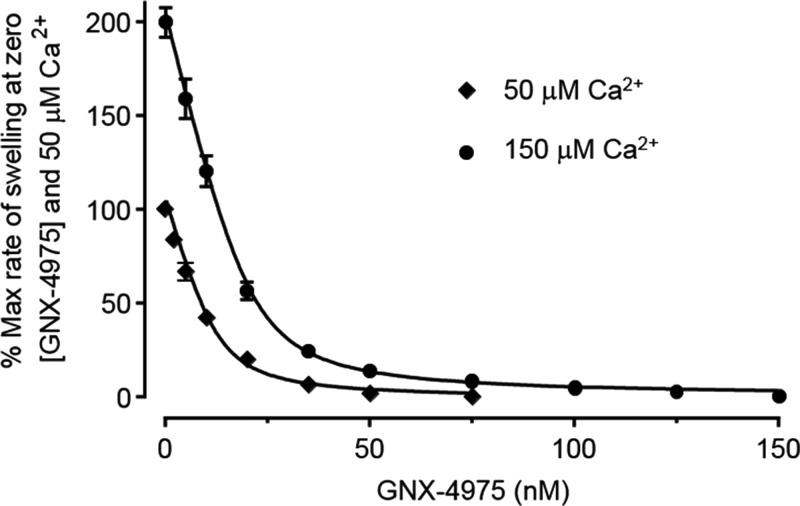
The concentration of Ca^2+^ used to initiate MPTP opening affects the number of GNX-4975-binding sites Experiments were performed exactly as described for Figure 1, but at a single protein concentration (1 mg/ml) and using either 50 or 150 μM Ca^2+^ to initiate swelling. The initial rate of swelling induced by 50 μM Ca^2+^ in the absence of GNX-4975 was set at 100 and all other rates calculated relative to this. Data for five separate mitochondrial preparations were fitted as described in Figure 1 to derive mean values (±S.E.M.) of *E*_t_ at 50 and 150 μM Ca^2+^ of 13.2±2.4 and 20.9±1.6 pmol/mg respectively (*P*<0.05). Corresponding *K*_i_ values remained unchanged at 1.91±0.35 and 2.15±0.26 nM respectively.

Increased [Ca^2+^] within the mitochondrial matrix is a key trigger for MPTP opening and, under de-energized conditions, the extent of MPTP opening can be increased progressively with increasing [Ca^2+^] [12,32]. In Figure 4, we investigate the effect of increasing the [Ca^2+^] used to trigger MPTP opening on the number of GNX-4975-binding sites. When MPTP opening was triggered with 150 μM [Ca^2+^], initial rates of swelling were 200±7.9% of the rates at 50 μM [Ca^2+^] and the corresponding number of GNX-4975-binding sites increased from 13.2±2.4 to 20.9±1.6 pmol/mg (means ± S.E.M. for five separate mitochondrial preparations; *P*<0.05) without any significant change in *K*_i_ value (1.91±0.35 and 2.15±0.26 nM respectively). These data imply that [Ca^2+^] may trigger MPTP opening by inducing the open conformation to which GNX-4975 binds.

**Figure 5:**
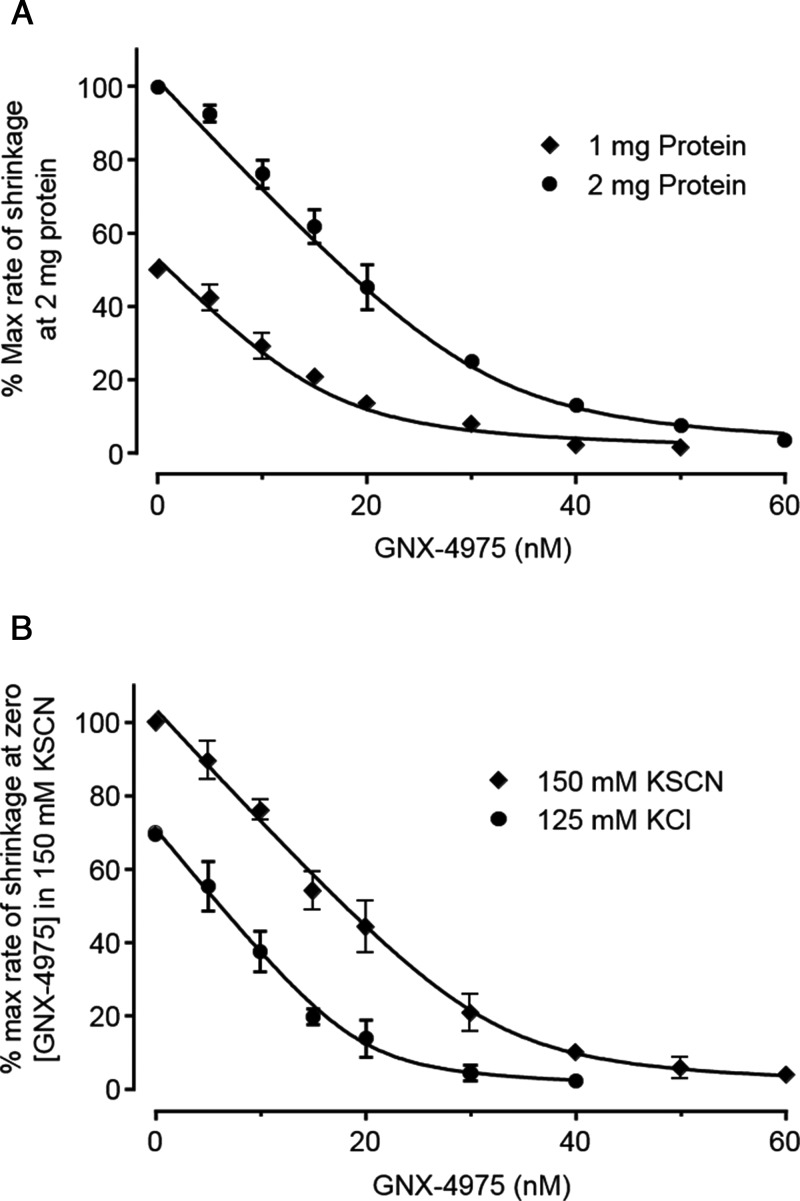
Assay of MPTP opening by shrinking of pre-swollen mitochondria confirms GNX-4975 to be a tight binding inhibitor and that activation of the MPTP by KSCN is reversible Pre-swollen mitochondria (1 or 2 mg) were incubated with increasing concentrations of GNX-4975 for 1 min in a cuvette containing 3 ml of KSCN or KCl medium. Ca^2+^ (50 μM) was then added and, after a further 1 min, shrinkage was initiated by addition of 500 μl of 50% (w/v) PEG 2000 followed by rapid mixing. Initial rates of increase in *A*_520_ were determined as described in the Experimental section. In (**A**), data are presented as means ± S.E.M. (error bars) for four separate mitochondrial preparations used at either 1 or 2 mg of protein per cuvette as indicated. Rates of swelling are expressed as a percentage of the zero inhibitor rate for each protein concentration and then values for 1 mg of protein are expressed as 50% of those at 2 mg of protein. This overcomes the lack of linearity of *A*_520_ with protein concentrations when fitting the data to the tight binding inhibitor equation using both sets of data. The mean derived values for the number of inhibitor binding sites and their *K*_i_ value were 46.9±5.3 pmol/mg and 2.08±0.30 nM respectively. In (**B**), pre-swollen mitochondria (2 mg total) were incubated in a KSCN or KCl medium, as indicated, containing the required concentrations of GNX-4975 and Ca^2+^ (50 μM) was added before initiating shrinkage by addition of PEG 2000. All initial rates of shrinking are expressed as a percentage of the rate in KSCN medium in the absence of inhibitor. Data are shown as means ± S.E.M. (error bars) for three separate mitochondrial preparations and were fitted as above. The derived values (±S.E.M. for the fit shown) for the number of inhibitor-binding sites in KSCN and KCl assay media were 49.0±2.8 and 29.6±2.3 pmol/mg respectively (*P* < 0.01) with corresponding *K*_i_ values of 1.13±0.37 and 0.811±0.34 nM respectively.

When swelling was employed to measure the extent of MPTP opening, the assay is initiated by addition of Ca^2+^. This places limitations on studying the interaction between [Ca^2+^] and GNX-4975 binding since the inhibitor must always be added before or at the same time as Ca^2+^. This limitation can be overcome by using a different assay of MPTP opening in which mitochondria are pre-swollen with Ca^2+^ and then caused to shrink by addition of PEG 2000 that is too large to permeate the MPTP. The PEG exerts an osmotic pressure on the IMM that shrinks the mitochondria at a rate (measured as an increase in *A*_520_) proportional to the extent of MPTP opening [40,41]. In this assay, the MPTP is opened by addition of Ca^2+^ prior to addition of PEG 2000 which allows GNX-4975 to be added either before or after Ca^2+^ prior to determination of the extent of MPTP opening. We first demonstrated that the shrinkage assay could be used to determine the number and *K*_i_ of GNX-4975-binding sites on the active MPTP. Data are presented in Figure 5A where the concentration-dependence of MPTP inhibition by GNX-4975 at two protein concentrations is determined using this assay. The data were fitted in exactly the same manner as the swelling data of Figure 1 and gave values of the number of binding sites for GNX-4975 of 46.9±5.3 pmol/mg with a *K*_i_ of 2.08±0.30 nmol (mean ± S.E.M. for three separate experiments). The number of GNX-4975-binding sites is substantially higher than that observed in the swelling data of Figure 1 (12.6±1.7 pmol/mg), and this probably reflects the formation of additional active MPTP complexes during the pre-swelling of mitochondria induced by addition of 1 mM [Ca^2+^] in KSCN buffer which is accompanied by the loss of intramitochondrial ATP and ADP, both of which inhibit MPTP opening [12,13]. Importantly, however, the *K*_i_ value for GNX-4975 was very similar to that obtained in the swelling assay (1.95±0.17 nM).

We also used the shrinkage assay to compare the concentration-dependence of GNX-4975 inhibition of MPTP opening in binding in KCl and KSCN buffers to see whether the activation by KSCN and increase in GNX-4975-binding sites seen in the swelling assay (Figure 2A) was also observed in the shrinkage assay. In all cases, the initial 20 min of pre-swelling was carried out in KSCN buffer and the mitochondria were maintained in KSCN buffer after their isolation by centrifugation. However, the mitochondria were then resuspended in the appropriate buffer (KSCN or KCl) for the shrinkage assay. Data are shown in Figure 5B and, in agreement with the swelling data (Figure 2A), the number of GNX-4975-binding sites (pmol/mg; mean ± S.E.M. for three separate preparations) increased from 29.6±2.3 in the KCl buffer to 49.0 ± 2.8 in KSCN buffer (*P*<0.01) without a significant change in *K*_i_ (0.81±0.34 and 1.13±0.37 nM respectively).

**Figure 6:**
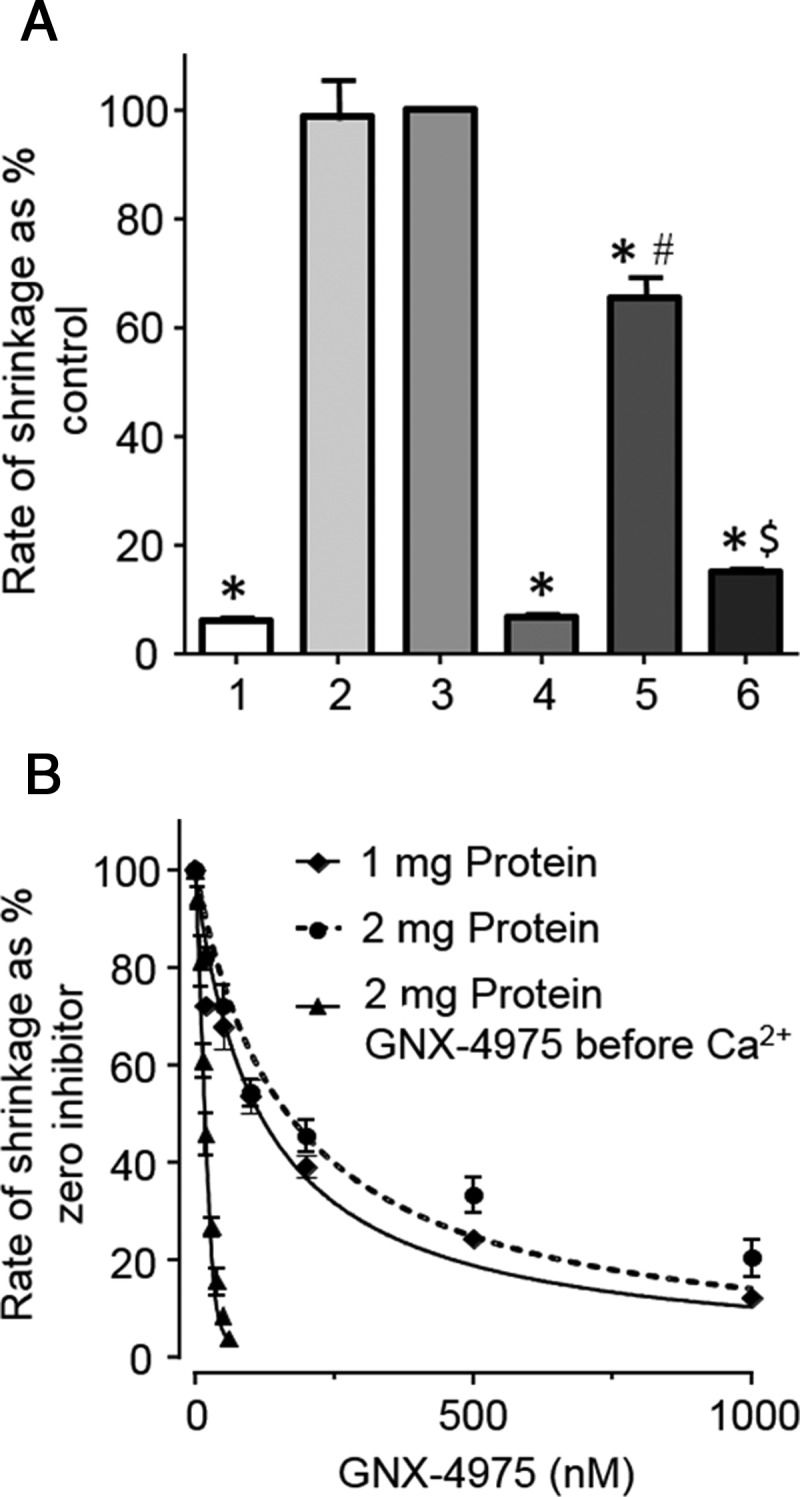
When GNX-4975 is added after Ca^2+^ its *K*_i_ for inhibition of MPTP opening is greatly reduced In (**A**), prior to initiation of shrinkage with PEG 2000, pre-swollen mitochondria (2 mg total) were incubated in KSCN medium without [Ca^2+^] (1), with 50 μM Ca^2+^ for 1 min (2) or 2 min (3) with 0.1 μM GNX-4975 for 1 min and then 50 μM Ca^2+^ for 1 min (4), with 50 μM Ca^2+^ for 1 min and then 0.1 μM GNX-4975 for 1 min (5) or both 50 μM Ca^2+^ and 0.1 μM GNX-4975, added simultaneously, for 2 min (6). Initial rates of swelling were expressed as a percentage of rates obtained with 2 min Ca^2+^ only (3) and as mean values (±S.E.M., error bars) for six separate mitochondrial preparations (*P<0.001 in comparison with data for condition 2; ^#^*P*<0.001 compared with conditions 4 and 5; ^$^*P*<0.001 compared with condition 4). In (**B**), pre-swollen mitochondria (1 or 2 mg total, as indicated) were incubated in 3 ml KSCN medium with 50 μM Ca^2+^ for 1 min followed by increasing concentrations of GNX-4975 prior to initiation of shrinkage with PEG 2000. Initial rates of shrinkage, expressed as a percentage of the rate in the absence of inhibitor, are shown as means ± S.E.M. (error bars) of three separate mitochondrial preparations and were fitted to a conventional hyperbolic inhibition curve (see ‘Experimental’). The derived *K*_i_ values (±S.E.M. for the fit shown) were 117±15 and 166±29 nM for 1 and 2 mg of protein respectively. For comparison, data from Figure 5B (closed triangles) have been included to illustrate that GNX-4975 is much more effective when added before Ca^2+^.

Having validated the shrinkage assay for measurement of GNX-4975 binding to the MPTP, we employed it to see whether the order of addition of Ca^2+^ and GNX-4975 affected the potency of the inhibitor. The data of Figure 6A show that when 0.1 μM GNX-4975 was added to pre-swollen mitochondria after 50 μM Ca^2+^, it was much less potent at inhibiting MPTP opening (34±3.8% inhibition) than when added before Ca^2+^ (93±0.4% inhibition). Even when added simultaneously with 50 μM Ca^2+^ there was some reduction in inhibition (85±1.0% inhibition). In Figure 6B, we compare the concentration dependence of GNX-4975 inhibition when added before or after 50 μM Ca^2+^. In the former case, the number of GNX-4975-binding sites and their *K*_i_ were determined in Figure 5B as 49.0±2.8 pmol/mg and 1.13±0.37 nM respectively (data are means ± S.E.M. for three separate experiments). However, when Ca^2+^ was added prior to the GNX-4975, the potency of the inhibitor was far less and independent of protein concentration. The data could now be fitted to the normal equation for inhibition (rate as % control=100/(1+[I]/*K*_i_)) with the derived *K*_i_ values at 1 and 2 mg of total protein being 117±15 and 166±29 nM respectively (data are means ± S.E.M. for three separate experiments; *P*>0.05).

### GNX-4975 binds only to the active conformation of the MPTP

**Figure 7:**
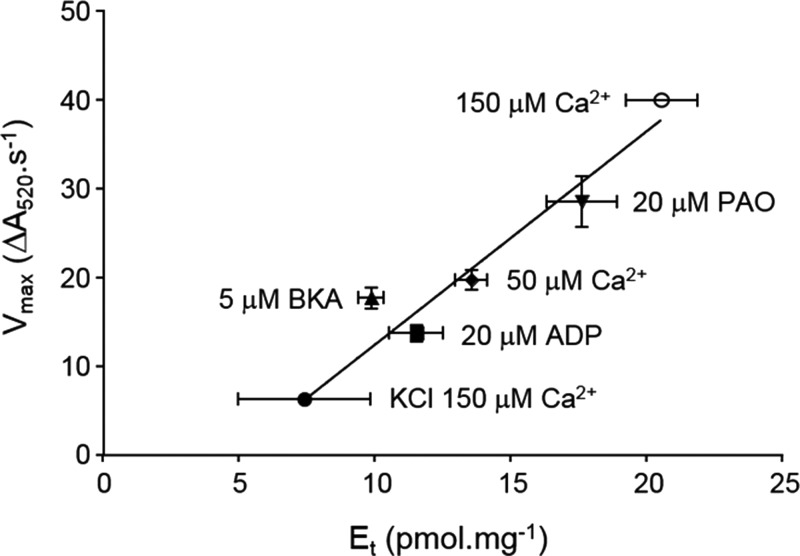
Control rates of swelling correlate with the calculated number of GNX-4975-binding sites as MPTP activity is increased For each condition shown in Table 1, absolute rates of swelling were calculated and plotted from the derived values of *E*_t_ and the rate constant *k*. Mean values were then calculated (±S.E.M. as vertical error bars) and plotted against the mean values of *E*_t_ (±S.E.M. as horizontal error bars). A total of 55 different data points are represented and were used to calculate a Pearson's correlation coefficient (*r*) of 0.743 (*P*<0.001).

In Table 1, we present data for the calculated number of GNX-4975-binding sites and the associated *K*_i_ values under all the conditions used in the present study. Reassuringly, differences in the calculated *K*_i_ values are small. We confirmed that these differences in the calculated *K*_i_ values could not account for any differences in calculated *E*_t_ values by re-analysing the data for each experimental condition using a fixed value of *K*_i_ (1.8 nM–the mean of the values obtained in the swelling experiments). These data are also included in Table 1. In Figure 7, we plot the calculated mean number of GNX-4975-binding sites determined under each condition with the mean absolute rate of swelling determined under the same conditions and demonstrate a very significant linear correlation (two-tailed Pearson's correlation coefficient (*r*) of 0.743; *P*<0.001). This strongly supports the hypothesis that GNX-4975 binds to the open pore complex and that a variety of agents that enhance or reduce MPTP opening do so by increasing or decreasing this active conformation of the MPTP. By contrast, titration of MPTP opening with CsA always produces the same number of binding sites irrespective of the degree of activation because it inhibits by interacting with CyP-D in its native conformation, preventing its binding to the membrane component(s) of the MPTP [28,29,41]. Since the number of GNX-4975-binding sites varies according to the activation state of the MPTP, it seems probable that this reflects a small fraction of a membrane protein (or proteins) undergoing a conformation change to form the pore.

**Table 1:** Collated *E*_t_ and *K*_i_ values for GNX-4975 binding The concentration of GNX-4975-binding sites (*E*_t_ in pmol/mg) and their *K*_i_ values (nM) are taken from the figures indicated. Where data for a particular condition are available from several figures (such as for KSCN buffer, 50 μM Ca^2+^), the value presented is the mean ± S.E.M. for all of the mitochondrial preparations used. The ‘Conditions’ column indicates whether the data are from the swelling or shrinkage experiment, the buffer and [Ca^2+^] used and the concentration of any MPTP modulators (if present). In the right-hand column, the data presented show the number of GNX-4975-binding sites (*E*_t_ in pmol/mg) calculated with a fixed *K*_i_ that is derived from the mean of all *K*_i_ values calculated under the different swelling and shrinkage conditions (1.80±0.079, *n*=61).

Conditions	Figure	*n*	*K*_i_ (nM)	*E*_t_ (pmol/mg)	*E*_t_ (pmol/mg) at fixed *K*_i_
Swelling assay					
KCl, 150 μM Ca^2+^	2A	5	1.68±0.13	9.43±1.4	9.10±1.5
KSCN, 50 μM Ca^2+^, 5 μM BKA	3A	6	1.46±0.16	9.43±0.47	8.56±1.7
KSCN, 50 μM Ca^2+^, 20 μM ADP	3B	6	1.45±0.15	11.0±0.97	10.1±1.1
KSCN, 50 μM Ca^2+^	2A, 2B, 3A, 3B, 4	23	1.71±0.13	13.1±0.60	12.9±0.59
KSCN, 50 μM Ca^2+^, 20 μM PAO	2B	6	2.56±0.14	17.2±1.3	19.5±1.6
KSCN, 150 μM Ca^2+^	2A	10	2.22±0.15	20.1±1.3	21.4±1.4
Shrinkage assay					
KCl, 50 μM Ca^2+^	5B	3	0.81±0.34	29.6±2.3	22.1±3.8
KSCN, 50 μM Ca^2+^	5B	3	1.13±0.37	49.0±2.8	45.7±5.2

### GNX-4975 stabilizes an interaction between the ANT and the PiC in heart IMM membranes

**Figure 8:**
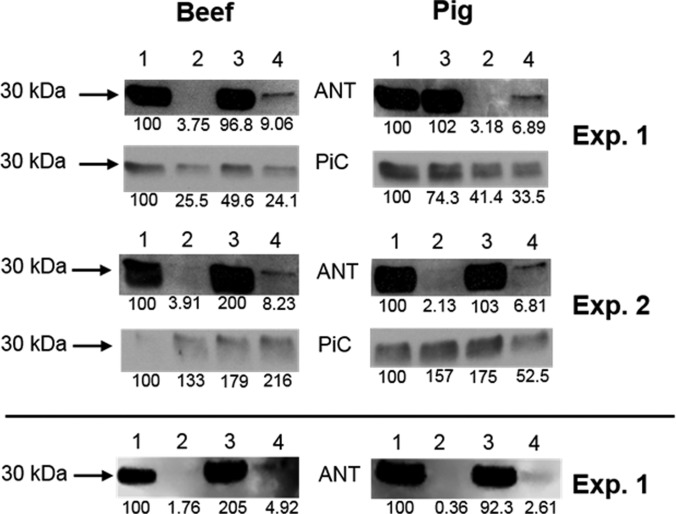
The effects of GNX-4975 on the binding to immobilized PAO of the ANT and PiC from control and CAT-treated heart mitochondria Binding of solubilized beef and pig heart IMM proteins to immobilized PAO was carried out as described in the Experimental section. Identical amounts of solubilized IMMs were added to the immobilized PAO in each case and specifically bound proteins were eluted using DTT and analysed by Western blotting using ANT- and PiC-specific antibodies as indicated. Only the section of the blot of ∼30 kDa is shown since no other significant bands were detected. Two separate experiments are shown for both beef and pig mitochondria and the values beneath each blot represent the integrated optical density for each band visualized on film and scanned. This was determined as described in the Experimental section and expressed as a percentage of the value for the untreated sample. The bottom blots represent samples from Experiment 1 re-analysed using an ODDESEY Fc (LI-COR) to directly detect the ECL signal. Lanes are: (1) untreated, (2) 1 μM GNX-4975 treated, (3) 10 μM CAT treated, (4) treated with 10 μM CAT followed by 1 μM GNX-4975.

We have suggested previously that both the ANT and PiC may be implicated in MPTP formation, with the pore forming at the interface of these two proteins in a novel conformation [1]. If this were the case, then GNX-4975 might bind at this interface and stabilize the interaction between the two proteins. In order to investigate this possibility, we made use of the observation that, for solubilized beef heart IMMs, both proteins bind to immobilized PAO. Binding of both proteins is prevented by the ubiquinone (UQ) analogues, UQ_0_ and Ro 68-3400, that inhibit MPTP opening, whereas binding of the ANT is specifically prevented by CAT that traps the ANT in its ‘c’ conformation [10,12,42]. Since CAT is associated with enhanced MPTP opening, it would be predicted that some ANT might remain bound to the PAO column in the presence of GNX-4975 to stabilize its interaction with the PiC. The data of Figure 8 confirm this to be the case for solubilized IMMs from both pig and beef heart mitochondria. As expected, treatment of heart mitochondria with 10 μM CAT prior to IMM isolation and solubilization totally prevented ANT binding to the PAO column. However, when either beef or pig heart mitochondria were treated with 10 μM CAT followed by 1 μM GNX-4975 prior to IMM isolation and solubilization, some ANT remained bound to the PAO column, consistent with the inhibitor stabilizing an interaction between the ‘c’ conformation of the ANT and the PiC or another IMM protein bound to the column. Underneath each blot, we give the integrated optical density for each band, calculated as the product of the ‘area’ and ‘mean grey value’ with appropriate background subtraction as described in the Experimental section, which reveals that the amount of the ANT bound after CAT treatment in the presence of GNX 4975 as a percentage of the total ANT bound in the absence of CAT is 4.51±0.33% (mean ± S.E.M., *n*=4 comprising the two beef and two pig experiments shown). This value is likely to be an overestimate because the amount of ANT remaining after CAT in the presence of GNX 4975 is very small and thus requires a long film exposure to visualize the band. This causes the intensity of the ANT band in the absence of CAT to exceed the linear response of the film. Indeed, in the bottom panel, we have re-analysed the blots for one set of pig and one set of beef mitochondria using an ODDESEY Fc (LI-COR) to directly detect the ECL signal. These data suggest that the overestimate is by a factor of ∼1.65 in both the pig and beef samples. From these data, we can estimate the amount of ANT whose binding is enhanced by GNX-4975 to be ∼2.7% of the total.

In order to establish whether other proteins that have been proposed to be part of the MPTP, such as subunits of the F_1_F_o_-ATP synthase, might act as a binding partner for the ANT, we performed a proteomic analysis using Orbitrap MS to identify all IMM proteins from beef heart mitochondria that bound to the PAO column. Full data are provided as a Supplementary Excel file but Table 2 presents the 12 most abundant proteins (each >1% of total protein bound) and, in addition, those components of the F_1_F_o_-ATP synthase that were detected (α, β, γ, b, oligomycin-sensitivity-conferring protein (OSCP), d and δ subunits). As reported previously, the dominant proteins were the ANT (11.8%) and PiC (4.1%), whereas the F_1_F_o_-ATP synthase subunits bound at levels similar to a range of other IMM proteins (<0.3%). This makes them less attractive candidates for the MPTP than the PiC and ANT and very unlikely targets for the site of PAO activation on the pore.

**Table 2:** Orbitrap MS analysis of proteins specifically eluted from immobilized PAO Binding of solubilized bovine heart mitochondria to immobilized PAO was carried out as described in the Experimental section. Specifically bound proteins were eluted using DTT, run 1 cm into a 12% acrylamide gel and analysed by Orbitrap MS as outlined in the Experimental section. The protein area column uses the three peak method of quantification to determine the relative amount of each protein in the sample. To calculate each individual protein contribution towards the total eluted protein as a percentage, each protein area score was standardized to the sum of all area scores for all of the protein hits eluted (295.43). Data for all 664 proteins detected are available online in a Supplementary Excel file.

Molecular mass (kDa)	Description	Protein score	Percentage of total protein
Most abundant proteins			
32.9	ADP/ATP translocase 1	34.78	11.77
35.0	Phosphate carrier protein	11.97	4.05
111.3	2-Oxoglutarate dehydrogenase	8.11	2.75
9.3	NADH dehydrogenase (ubiquinone) 1 α subcomplex subunit 4	7.34	2.48
47.9	Keratin, type I cytoskeletal 18	7.31	2.47
36.7	NADH dehydrogenase (ubiquinone) 1 α subcomplex subunit 10	7.12	2.41
36.7	NDUFA10 protein (fragment)	5.49	1.86
33.2	D-β-Hydroxybutyrate dehydrogenase	5.14	1.74
63.1	Keratin, type II cytoskeletal 1	4.73	1.60
68.7	Stress-70 protein	4.70	1.59
31.6	Voltage-dependent anion-selective channel protein 2	4.20	1.42
47.2	Creatine kinase S-type	4.16	1.41
ATP synthase subunits			
55.2	ATP synthase subunit α	0.86	0.29
51.6	ATP synthase subunit β	0.81	0.27
30.3	ATP synthase subunit γ	0.36	0.12
24.7	ATP synthase subunit b	0.26	0.09
20.9	ATP synthase subunit OSCP (oligomycin-sensitivity-conferring protein)	0.22	0.08
18.7	ATP synthase subunit d	0.19	0.06
15.1	ATP synthase subunit δ	0.14	0.05

## DISCUSSION

The exact molecular composition of the membrane component of the MPTP remains uncertain with evidence presented for a regulatory or structural involvement of the ANT, PiC, different components of the F_1_F_o_-ATP synthase and, most recently, the inner membrane AAA protease SPG7 [1,4,6,7,25,26]. Irrespective of the exact composition of the MPTP, it seems unlikely that any of these proteins form the pore in their normal conformation since their concentrations are far higher than the number of GNX-4975-binding sites determined here (9–50 pmol/mg of mitochondrial protein depending on the degree of activation). Thus the concentration of ANT in rat liver and bovine heart mitochondria is 0.3 and 1.2 nmol/mg respectively [43], whereas for the PiC values of 0.28 and 0.67 nmol/mg have been estimated [44]. The concentration of the F_1_F_o_-ATP synthase is ∼0.2 nmol/mg in rat liver mitochondria [45] and for CyP-D values of ∼100–200 pmol/mg in rat liver and heart mitochondria have been estimated [28,46].

### GNX-4975 binds only to the active MPTP at the Ca^2+^ activation site

Our data demonstrate that GNX-4975 is a potent MPTP inhibitor (*K*_i_ ∼ 1.8 nM) that binds only to the active (open) form of the pore. The data, summarized in Table 1 and Figure 7, show that activators of the MPTP such as PAO, KSCN and higher [Ca^2+^] increase the number of inhibitor-binding sites, whereas the BKA and ADP that reduce MPTP opening decrease the number of GNX-4975-binding sites. None of these effectors caused a major change in the *K*_i_ of GNX-4975 for inhibition of MPTP opening provided the inhibitor was added before Ca^2+^ as in the swelling assay for MPTP opening. However, using the shrinkage assay, it was shown that if Ca^2+^ was added prior to GNX-4975 the *K*_i_ was greatly elevated (Figure 6). The simplest explanation for these data would be that [Ca^2+^] and GNX-4975 compete for the same binding site on the active conformation of the MPTP, but that once GNX-4975 is bound its dissociation is so slow that Ca^2+^ cannot displace it within the timeframe of the experiment. Such behaviour is well documented for tight binding inhibitors [47], and a low off rate constant (*k*_off_) has also been observed for CsA binding [41,48]. The *k*_off_ could be determined from the *K*_i_ if the rate constant for inhibitor binding (*k*_on_) was known, since *K*_i_=*k*_off_/*k*_on_. Values of *k*_on_ for tight inhibitor binding to soluble enzymes have been shown to range between 10^4^ and 10^9^ mol^−1^·l^−1^·s^−1^ and can be substantially lower where inhibitor binding produces a conformation change [47]. For a *k*_on_ of 10^5^ mol^−1^·l^−1^·s^−1^ the calculated *k*_off_ is 2×10^−4^ s^−1^ implying that it would take nearly 1 h for 50% dissociation of the inhibitor and thus that Ca^2+^ would not displace the GNX-4975 during the time of the assay. This would explain the low *K*_i_ values observed when the inhibitor was added prior to Ca^2+^, but considerably higher values when the order of addition was reversed.

### The open MPTP is formed when a small fraction of an IMM protein adopts a novel conformation

Although CsA and GNX-4975 exhibit similar *K*_i_ values for inhibition of the MPTP, they act quite differently. CsA binds to CyP-D and prevents its association with a membrane component of the MPTP that facilitates the calcium-activated conformational change required for pore opening [1,4,6,25]. As such the number of CsA-binding sites determined from inhibitor titrations of MPTP opening always matches the amount of CyP present in the mitochondrial matrix [28,29]. Conversely, for GNX-4975 the number of binding sites varies depending on the activity of the MPTP. This can be explained if formation of the active MPTP requires a fraction of a native IMM protein (or proteins) to adopt a novel conformation to which GNX-4975 binds. The concentration of GNX-4975-binding sites determined in liver mitochondria varied between 9 and 20 pmol/mg of protein depending on conditions in normal de-energized liver mitochondria, and increased up to ∼50 pmol/mg of protein in pre-swollen mitochondria (Table 1). Thus the IMM component(s) that form the MPTP must be present in concentrations significantly higher than this which is true of the proposed candidate proteins, the PiC, ANT and subunits of the F_1_F_o_-ATP synthase as noted above.

### GNX-4975 stabilizes an interaction between the PiC and the ANT in its ‘c’ conformation

We have previously proposed that the MPTP may be formed at the interface between the ANT and the PiC when one or both of these proteins is in a distinct conformation such as the ‘c’ conformation of the ANT and the PiC modified by oxidative stress or PAO [1]. This would be entirely consistent with the GNX-4975-binding studies discussed above and received further support from the effects of CAT and GNX-4975 on the binding of IMM proteins to immobilized PAO shown in Figure 8. We have previously shown that PAO activates the MPTP by binding to vicinal thiol groups on both the ANT and PiC [10,12,37]. If the MPTP is formed when such an interaction occurs between the PAO-bound PiC and the ANT in the ‘c’ conformation, then a small fraction of the ANT might remain attached to the PiC on the column if this interaction survived the solubilization process. This was observed in the presence of GNX-4975 but not in its absence (Figure 8 and Table 2), suggesting that inhibitor binding may stabilize the interaction between the two proteins. The relatively small amount (2.7%) of the ANT bound compared with the total ANT bound in the absence of CAT is to be expected since the GNX-4975-binding data suggest only ∼10–20 pmol/mg of protein of the active MPTP complex compared with a concentration of the ANT of 300 and 1200 nmol/mg in rat liver and bovine heart mitochondria respectively [43]. Other proteins that have been proposed to be important in MPTP formation, such as subunits of the F_1_F_o_-ATP synthase [4,7] and the mitochondrial AAA protease SPG7 [26], were either not detected in the protein fraction bound to the PAO or were present at extremely low levels (<0.3%) compared with the ANT and PiC (11.8 and 4.1% respectively) as shown in Table 2. Although this does not rule out their role in MPTP formation, it does suggest that these proteins are unlikely to contain the vicinal thiol groups whose binding of PAO activates the MPTP.

### Insights into the molecular mechanism of the MPTP

**Figure 9:**
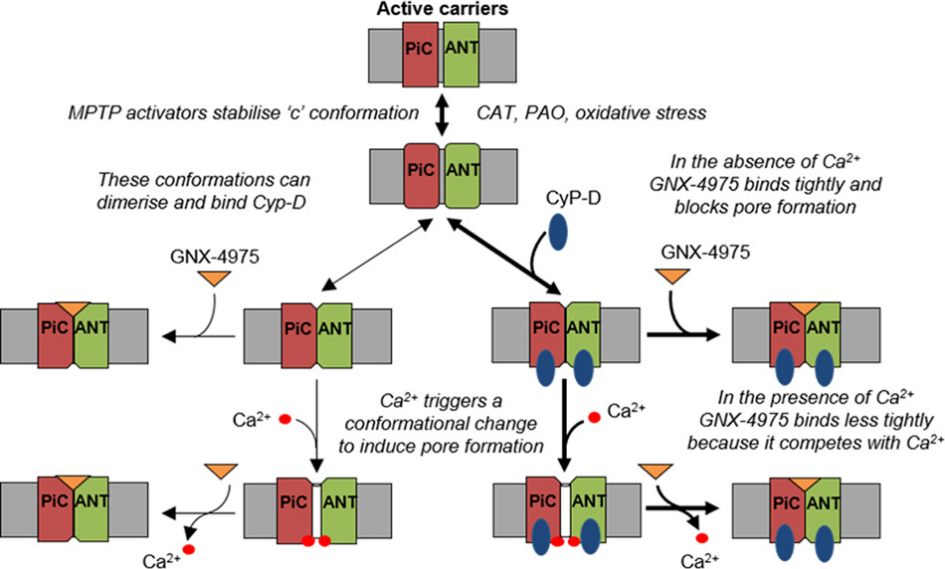
A schematic diagram showing a plausible mechanism for the MPTP and its inhibition by GNX-4975 In their cytosol-facing (‘c’) conformation, such as when stabilized by CAT or oxidative stress, IMM candidate proteins for the MPTP such as the ANT and PiC can undergo a further conformation change that is enhanced by KSCN and P_i_ and to which CyP-D can bind. This produces an interface between the two proteins, perhaps involving cardiolipin that represents the MPTP in its closed state. Binding of Ca^2+^ on the matrix face triggers a further conformation change, facilitated by CyP-D (thick arrows cf. thin arrows), to open the MPTP. GNX-4975 binds to the closed MPTP, stabilizing this conformation and so preventing Ca^2+^ from triggering pore opening which is reversible. The equilibrium between open and closed states is determined by the [Ca^2+^] whose action is competitively inhibited by GNX-4975 binding. However, the rate of dissociation of GNX-4975, once bound, is very low and thus not readily overcome by increasing [Ca^2+^].

The data we have obtained with GNX-4975 are entirely consistent with previous data from our laboratory, reviewed in [1], that led us to propose that such conformational changes in both the ANT and PiC may induce an interface between these two membrane proteins, perhaps involving their tightly bound annular cardiolipin and co-ordinated by the F_1_F_o_-ATP synthase in the proposed ATP synthasome complex. The ANT provides the bin-ding site for regulation by adenine nucleotide, ligands of the ANT and oxidative stress as has been demonstrated by the loss of such regulation in mitochondria lacking ANT1 and ANT2 [17]. Furthermore, the reconstituted ANT can form Ca^2+^-activated channels that are regulated by CyP-D [16,49]. Involvement of the PiC can account for the activation of the MPTP by P_i_ and its inhibition by low concentrations of *N*-ethylmaleimide, as well as providing an additional site of action of oxidative stress and PAO [10,14]. We have further proposed that Ca^2+^ may act through its binding to annular cardiolipin at the interface between the PiC and the ANT [1]. Indeed, cardiolipin is one of the few calcium-binding molecules that can distinguish between Sr^2+^ and Ca^2+^ [50] which may explain why the MPTP is activated by Ca^2+^, but not Sr^2+^ [40]. Our present data suggest that GNX-4975 binds to the active form of the MPTP in competition with Ca^2+^ at this interface. This is illustrated schematically in Figure 9.

Despite this evidence in favour of our model, it cannot be ignored that knockdown of either the ANT or the PiC only attenuates MPTP activity and modulates its regulatory properties. These data demonstrate that neither the PiC nor the ANT is indispensable for MPTP activity, implying that other proteins can form the MPTP. However, the PiC and ANT are members of the 53-strong mitochondrial carrier family (MCF) which share a common structure in which six transmembrane helices form the substrate translocation pathway that involves conformational changes between inward- and outward-facing conformations [51]. Thus it is possible that any member of the MCF can enter a conformation in which they form an interface with another MCF member to form the MPTP. Annular cardiolipin is likely to surround all MCF members and thus could provide a common Ca^2+^-binding site whatever MCF members form the pore. Nevertheless, because the ANT and PiC are by far the most prevalent MCF members and may already be in close association through the ATP synthasome, they will usually form the MPTP and account for its regulation by adenine nucleotide, ANT ligands, PAO, oxidative stress and P_i_ [1].

## Abbreviations

ANT: adenine nucleotide translocase
BKA: bongkrekic acid
CAT: carboxyatractyloside
CsA: cyclosporin A
CyP-D: cyclophilin D
IMM: inner mitochondrial membrane
ISB: isolation buffer
MCF: mitochondrial carrier family
MPTP: mitochondrial permeability transition pore
NTA: nitrilotriacetic acid
PAO: phenylarsine oxide
PCB: PAO column buffer
PEG: polyethylene glycol
PiC: phosphate carrier
SPG7: spastic paraplegia 7
